# The Human Penis Is a Genuine Immunological Effector Site

**DOI:** 10.3389/fimmu.2017.01732

**Published:** 2017-12-14

**Authors:** Alexis Sennepin, Fernando Real, Marine Duvivier, Yonatan Ganor, Sonia Henry, Diane Damotte, Marc Revol, Sonia Cristofari, Morgane Bomsel

**Affiliations:** ^1^Laboratory of Mucosal Entry of HIV-1 and Mucosal Immunity, Department of Infection, Immunity and Inflammation, Cochin Institute, INSERM, Paris, France; ^2^CNRS, UMR8104, Paris, France; ^3^Paris Descartes University, Sorbonne Paris Cité, Paris, France; ^4^Anatomy and Pathological Cytology Service, GH Cochin-Saint Vincent de Paul, Paris, France; ^5^Plastic Surgery Service, Saint Louis Hospital, Paris, France

**Keywords:** mucosal immune system, male genial tract, mucosal immunity, B cells, mucosal vaccines

## Abstract

The human penis is a main portal of entry for numerous pathogens, and vaccines able to control resulting infections locally are highly desirable. However, in contrast to the gastrointestinal or vaginal mucosa, the penile immune system and mechanisms inducing a penile immune response remain elusive. In this descriptive study, using multiparametric flow cytometry and immunohistochemistry, we characterized mucosal immune cells such as B, T, and natural killer (NK) cells from the urethra, fossa, and glans of human adult penile tissues. We show that memory B lymphocytes and CD138^+^ plasma cells are detected in all penile compartments. CD4^+^ and CD8^+^ T lymphocytes reside in the epithelium and lamina propria of the penile regions and have mostly a resting memory phenotype. All penile regions contain CD56^dim^ NK cells surface expressing the natural cytotoxicity receptor NKp44 and the antibody-dependent cell cytotoxicity receptor CD16. These cells are also able to spontaneously secrete pro- and anti-inflammatory cytokines, such as IL-17 and IL-22. Finally, CCR10 is the main homing receptor detected in these penile cells although, together with CCR3, CCR6, and CCR9, their expression level differs between penile compartments. Unlike antigen-presenting cells which type differ between penile regions as we reported earlier, urethral, fossa, and glans content in immune B, T, and NK cells is comparable. However, median values per each analysis suggest that the glans, containing higher number and more activated NK cells together with higher number of terminally differentiate effector CD8^+^ T cells, is a superior effector site than the urethra and the fossa. Thus, the human penis is an immunologically active tissue containing the cellular machinery required to induce and produce a specific and effective response against mucosal pathogens. It can therefore be considered as a classic mucosal effector site, a feature that must be taken into account for the elaboration of efficient strategies, including vaccines, against sexually transmitted infections.

## Introduction

Sexually transmitted infections (STIs) are a main health issue worldwide (1), and the male genital tract is a portal of entry for numerous sexually transmitted pathogens, including viral and bacterial pathogens, such as human papilloma virus (HPV) (2), *Chlamydia trachomatis* (3) or *Neisseria gonorrhoeae* (4). Furthermore, we and others also demonstrated that human immunodeficiency virus type 1 (HIV-1) targets the penile foreskin and urethra (5–10).

To reduce or prevent these STIs, vaccine strategies targeting the penis are crucially needed. Accordingly, initial HIV-1 vaccine studies were able to induce HIV-1 specific mucosal antibodies, although non-neutralizing, in the male genital mucosa (11). Furthermore, exposed seronegative (ESN) men harbor high urethral concentrations of HIV-1-specific IgA induced by non-protected insertive sexual intercourses with seropositive female partners (12). These studies indicate that the human male genitals, as in other species (13), are effector sites. However, the lack of progress in developing vaccines to stimulate local protection in the penis is mainly due to the lack of information on the penile immune system.

The human penis consists of four different regions: (i) the foreskin, a stratified keratinized epithelium, with a highly keratinized outer face and a less keratinized inner one facing the glans (8), (ii) the glans, a stratified keratinized epithelium; (iii) the fossa navicularis (referred to here as fossa), a stratified non-keratinized epithelium, and (iv) the urethra, a pseudo-stratified non-keratinized epithelium (6, 8). The penis susceptibility to STI depends largely on the intrinsic characteristics of the mucosal immune system of each of these regions.

Innate and adaptive immune responses contribute both to protection at mucosal surfaces (14). The mucosal innate immune system is the first line of defense against mucosal pathogens and comprises numerous components including epithelial barriers, antimicrobials peptides (15), pattern recognition receptors, such as toll-like receptors (TLRs) (16), and inflammatory immune cells, such as natural killer (NK) cells and neutrophils, which are mainly involved in apoptosis of infected cells and phagocytosis, respectively. Antigen-presenting cells that include macrophages, Langerhans cells (LCs) and dendritic cells (DCs), participate in innate immune responses, as well as the initiation of adaptive immune responses by presenting antigens to lymphocytes. Such adaptive immune responses, which take place in a second step following the innate immune responses, are pathogen specific and involve two arms, namely, the humoral response coordinated predominantly by B cells, with or without CD4^+^ T cells help, and the cellular response driven by cytotoxic T cells.

Penile mucosal immune cells and their interactions with STI have been little studied due to the difficulty in obtaining human tissues, whereas the foreskin immunity is better understood particularly in the context of HIV-1 infection. Hence, we showed that HIV-1 targets first LCs during sexual transmission of HIV-1 in non-circumcised men (7), providing an explanation at the cellular level to the reduction by >60% of HIV-1 acquisition in men provided by removal of the foreskin following circumcision. Circumcision also protects men efficiently against other STI including HPV and herpes simplex virus (HSV)-2 (17). In agreement with an only partial protection to STI resulting from circumcision, other penile regions are targeted by STIs. Indeed, HIV-1 also targets macrophages in the penile urethra as we reported (10). Other studies (5, 6, 18) have reported on the immune cell content of the penis using qualitative morphological analyses, although a detailed phenotype and the role of these mucosal immune cell populations were not assessed. To fill this gap, we provide here an in-depth characterization of B, T, and NK cells present in the different penile regions, namely, the urethra, fossa, and glans mucosae, a crucial prerequisite for the elaboration of efficient preventive and vaccinal strategies against STIs.

## Materials and Methods

### Ethical Statement

The study was performed according to local ethical regulations, following approval by the local ethical committee [Comité de Protection des Personnes (CPP) Île-de-France XI, approval no. 11016]. Written informed consent was provided by all study participants.

### Tissues

Whole penile tissues were obtained from 39 individuals undergoing elective gender reassignment surgery at the Saint Louis Hospital in Paris, France (mean age 38 years, range 19–58 years). Hormonal treatment was stopped 2 months before surgery, and all patients lacked history of STIs during the 6 months before the surgery. Tissues were transported to the laboratory immediately after surgery in phosphate-buffered saline (PBS) supplemented with 20 μg/ml gentamicin (Gibco) and used within the next 2 h.

### Single Cell Suspensions

Urethra, fossa, and glans were first mechanically separated, each region cut into 8 mm × 8 mm pieces, and the underlying fat and muscle removed. To prevent denaturation or removal of cell surface molecules by dispase/trypsin enzymes that are routinely used to separate the epidermal and dermal compartments, cells were directly extracted from the tissues by collagenase type IV digestion (2 mg/ml, Sigma) in the presence of DNAse I (200 U/ml, Roche) for 2 h at 37°C (7, 9). After enzyme inactivation with RPMI medium containing 10% fetal bovine serum (FBS, Gibco), tissue pieces were vortexed for 30 s. The resulting cell suspensions were filtered through 100 μm nylon cell strainers (Falcon) and centrifuged for 5 min at 450 *g*.

### Flow Cytometry

Cells were resuspended in PBS-2% FBS, transferred to a 96 round-bottom well plate (10^6^ cells/well) and labeled with indicated combinations of antibodies (Table S1 in Supplementary Material) or matched isotype control antibodies (Table S2 in Supplementary Material) for 30 min at 4°C. Cells were washed twice in PBS-2% FBS and fixed with Cytofix/Cytoperm solution (BD Biosciences) for 15 min at room temperature.

**Figure 1:**
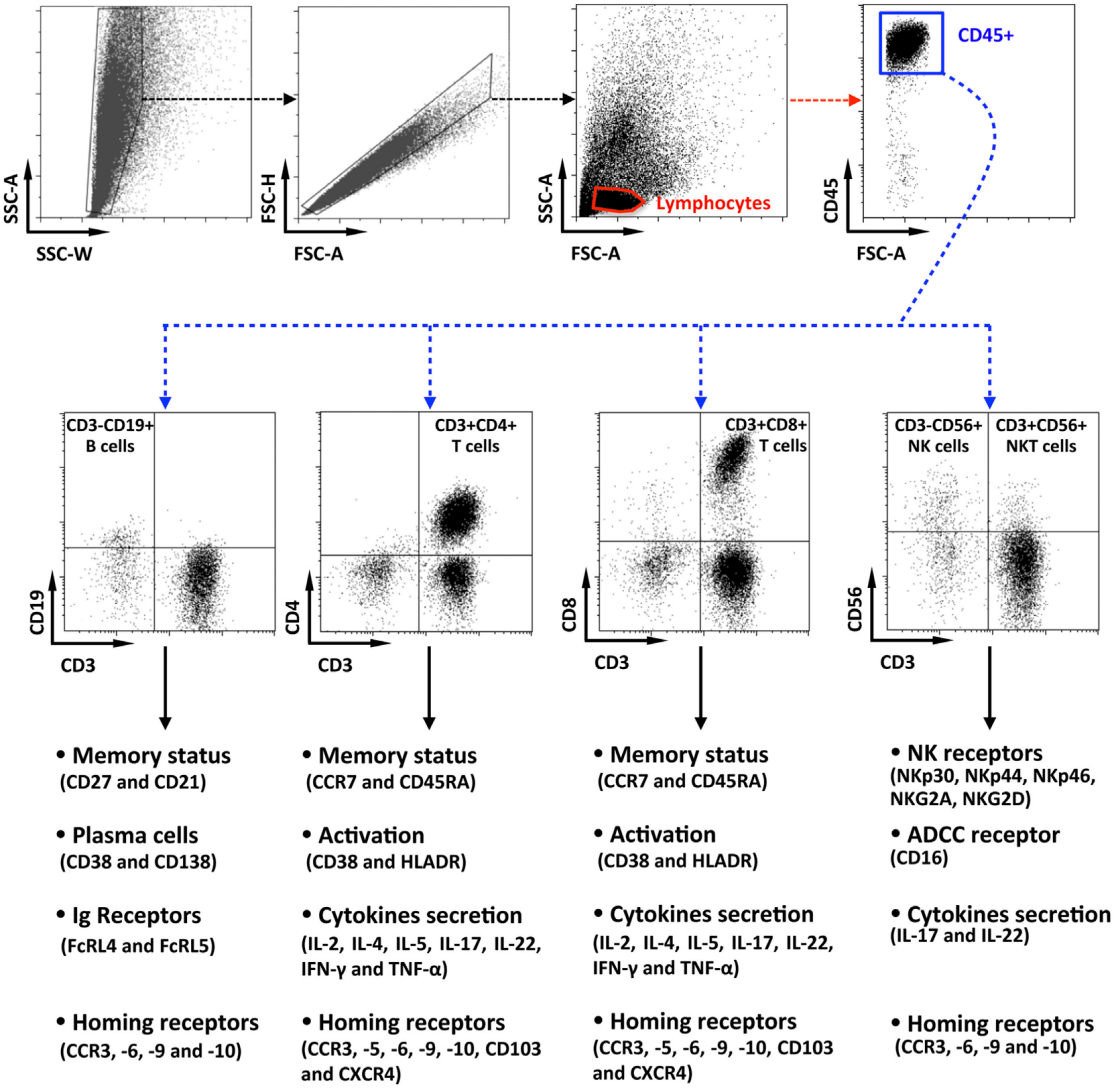
Flow cytometry gating strategy for the characterization of penile cells. Representative dot plots of penile single cells suspensions studied by multiparametric flow cytometry, showing the gating strategy. After cell-doublet exclusion (SSC-W/SSC-A and FSC-A/FSC-H dot plots), lymphocytes were gated based on their forward (FSC-A) and side (SSC-A) scatter (lymphocytes gate). After gating on CD45^+^ lymphocytes, penile immune cell populations were defined as CD3^−^CD19^+^ B cells, CD3^+^CD4^+^ T cells, CD3^+^CD8^+^ T cells, CD3^−^CD56^+^ natural killer cells, and CD3^+^CD56^+^ NKT cells. Each population was then studied for their phenotypes, activation status, function as well as for the presence of potential homing receptors according to the strategy indicated in the figure.

Data were acquired, with an LSRII cytometer (Becton-Dickinson, Cochin CYBIO platform) and analyzed with Kaluza Software (Beckman-Coulter). As shown on Figure 1, penile lymphocytes were identified based on their forward and side scatter, after a cell-doublet exclusion step. Next, lymphocytes were identified as CD45^+^ cells, and immune cell populations were defined according to their phenotypic characteristics, namely, CD3^−^CD19^+^ for B cells, CD3^+^CD4^+^ for CD4^+^ T cells, CD3^+^CD8^+^ for CD8^+^ T cells, and CD3^−^CD56^+^ or CD3^+^CD56^+^ for NK and NKT cells, respectively. Finally, phenotypes, activation status, as wells as functions, were evaluated for each population as indicated.

### Intracellular Cytokine Staining

Penile cells were plated in 500 µl culture medium (RPMI medium containing 10% FBS) for 5 h at 37°C in the presence of Brefeldin A (5 µg/ml, Sigma). Cells were washed twice in PBS-2% FBS and labeled with anti-CD4, anti-CD8 and anti-CD56 antibodies or matched isotype control antibodies for 30 min at 4°C. Cells were washed twice in PBS-2% FBS and fixed with Cytofix/Cytoperm solution (BD Biosciences) for 15 min at room temperature. Cells were washed with PermWash buffer (BD Biosciences) and stained for intracellular IL-2, IFN-γ, TNF-α, IL-4, IL-5, IL-17, and IL-22 for 30 min at 4°C using appropriate antibodies (Table S1 in Supplementary Material) or corresponding isotype control antibodies (Table S2 in Supplementary Material), diluted in PermWash buffer.

Data were acquired with an LSRII cytometer (Becton-Dickinson, Cochin CYBIO platform) and analyzed following the gating strategy shown in Figure 1 with Kaluza Software (Beckman-Coulter) as indicated before.

### Immunohistochemistry

Urethra, fossa, and glans tissue pieces were fixed with PBS-4% paraformaldehyde, embedded in paraffin and serially sectioned at 4 µm. Immunohistochemistry was performed as we previously described (7, 10) using appropriate antibodies (Table S3 in Supplementary Material), and the LSAB2 System HRP (Abcam) labeling kit, and visualized with diaminobenzidine (Dako) or histogreen (Abcys) peroxidase substrates according to the manufacturer’s recommendations. Image acquisition was performed with an OlympusBX63F microscope (Cochin IMAG’IC platform), and images were analyzed using the open-access GNU Image Manipulation Program (The GIMP Team).

### Cytokines/Chemokine Quantification

Urethra, fossa, and glans tissue pieces (5 mm × 3 mm × 1 mm) were lysed in 500 μl of PBS-0.2% SDS containing a protease inhibitor cocktail (1:1,000 dilution, Roche), disrupted using a Qiagen TissueRuptor. Resulting cell lysates were stored at −80°C. Cytokines, namely, MCP-1/CCL2, MIP-3α/CCL20, RANTES/CCL5, TRAIL/TNFSF10, MCP-4/CCL13, IL-4, IL-13, CTACK/CCL27, and Eotaxin/CCL11, were quantified using the Luminex technology (LXSAHM-12 Kit, R&D) on a Bio-Plex 200 (Bio-Rad, Cochin CYBIO platform) according to the manufacturer’s recommendations. Furthermore, CCL25 and CCL28 were quantified using the Quantikine immunoassay (DTK00 and DCC280 kits, respectively, R&D) according to the manufacturer’s recommendations.

### Statistical Analysis

Statistical analysis was performed with Prism6 software (GraphPad). For flow cytometry analyses, tissues of the various penile regions did not always originate from the same individual. The results are expressed as median, followed by the 95% confidence interval (95% CI). For comparing the immune cell populations between the different penile compartments, although such statistics have to be taken cautiously due to the limited number of tissue samples, the non-parametric Kruskal–Wallis test was used. Pairwise comparisons were performed by the non-parametric Mann–Whitney *U*-test. *p*-Values < 0.05 were considered significant. *p < 0.05, **p < 0.01, and ***p < 0.001.

## Results

### Memory B Cells Are Present in All Penile Regions

B cells are an important population of adaptive immunity driving the humoral response by producing antibodies after antigenic stimulation. Characterization of their phenotypes is a fundamental prerequisite for the elaboration of efficient vaccine strategies against STIs.

**Figure 2:**
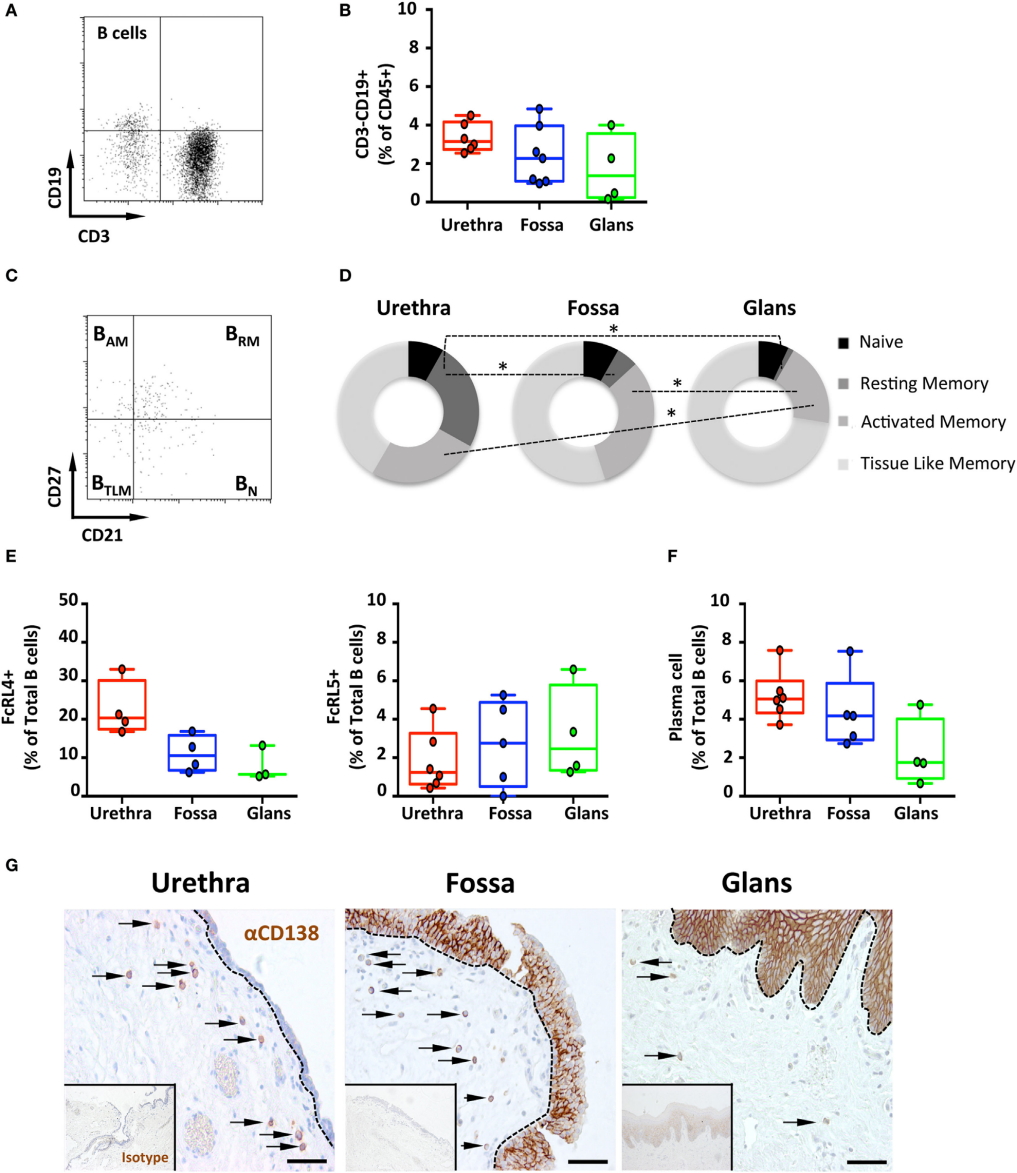
Frequency and phenotype of B cells subsets and plasma cells (PCs) distribution in the penile mucosa. **(A)** Representative dot plot and **(B)** box-and-whisker plots of total B cells proportions in urethra, fossa, and glans. B cells were characterized by a CD45^+^CD3^−^CD19^+^ phenotype [*n* = 6, 7, and 4 different donors for urethra, fossa, and glans, respectively; mean age 43 years (range: 24–58)]. **(C)** Representative dot plot and **(D)** proportions of memory B cell subsets in the different parts of penile mucosa [*n* = 6, 5, and 4 different donors for urethra, fossa, and glans, respectively; mean age 39 years (24–56)]. Memory phenotype was defined as follows: naive (B_N_, CD21^+^CD27^−^), resting memory (B_RM_, CD21^+^CD27^+^), activated memory (B_AM_, CD21^−^CD27^+^), and tissue-like memory cells (B_TLM_, CD21^−^CD27^−^) after gating on CD45^+^CD3^−^CD19^+^ B cell population. **(E)** Proportions of FcRL4^+^ [left panel, *n* = 4, 4, and 3 different donors for urethra, fossa, and glans, respectively; mean age 43 years (24–58)] and FcRL5^+^ [right panel, *n* = 6, 5, and 4 different donors for urethra, fossa, and glans, respectively; mean age 43 years (24–58)] cells in the total CD45^+^CD3^−^CD19^+^ B cell population in urethra, fossa, and glans. **(F)** Proportions of PCs in penile tissues [*n* = 6, 5, and 4 different donors for urethra, fossa, and glans, respectively; mean age 39 years (24–56)] after gating on CD45^+^CD3^−^CD19^+^CD38^+^CD138^+^ population. **(G)** Immunohistochemistry analysis of CD138^+^ PCs (brown), by comparison with isotype control (inset), in urethra, fossa, and glans [representative of *n* = 5 different donors for each tissue; mean age 41 years (26–54)]. Cells were stained with an anti-CD138 antibody or a rabbit IgG isotype control and revealed using diaminobenzidine peroxidase substrate (brown staining). Scale bar = 50 μm. All box-and-whisker plots represent minimum-to-maximum values, and each point corresponds to one donor. Statistical analyses were performed first by the Kruskal–Wallis test; pairwise comparisons were performed by the Mann–Whitney *U*-test. *p < 0.05, **p < 0.01, and ***p < 0.001.

Therefore, the presence of B lymphocytes, defined by the expression of the pan leukocyte marker CD45 and CD19, but lack of CD3, was determined in each penile compartment by multiparametric flow cytometry (Figures 2A,B). CD3^−^CD19^+^ B cells represented 0.5–3.0% of CD45 cells, with median [95% CI range]: 3.2% [2.8–3.45] in the urethra, 2.3% [1.7–2.8] in the fossa, and 1.3% [0.7–2.0] in the glans.

Next, co-expression of CD21 and CD27 was used to characterize the four B cell subsets (19), namely, CD21^+^CD27^−^ naïve (B_N_), CD21^+^CD27^+^ resting memory (B_RM_), CD21^−^CD27^+^ activated memory (B_AM_), and CD21^−^CD27^−^ tissue-like memory (B_TLM_) B cells (Figures 2C,D).

The major B cell subset was B_TLM_ in all three penile compartments. Although the frequency of B_TLM_ was similar between urethra (53.2% [43.3–63.1]) and fossa (64.5% [55.5–73.5]), it was higher in the glans (74.4% [70.9–77.8]). In addition, while B_AM_ and B_RM_ were both present in urethra (32.5% [27.9–37] and 31.9% [29.1–34.7], respectively) and fossa (37.4% [31.4–43.4], and 5.9% [3.7–8.1], *p* = 0.0286 vs urethra, respectively), B_AM_ were significantly less abundant (19.3% [15.4–23.2] *p* = 0.0236 relative to the urethra), and B_RM_ were almost absent (1.3% [0.85–1.85], *p* = 0.0286 relative to the urethra) in the glans. Finally, B_N_ were present at similar proportions in urethra (10.5% [8.6–12.4]), fossa (9.6% [7–12.2]), and glans (7.2% [5.8–8.5]).

Altogether, memory B cells (B_TLM_ > B_AM_) predominate over resting memory/naive B cells in the penis.

### Penile B Cells Express the IgA Fc Receptor-Like 4 (FcRL4) and the IgG Fc Receptor-Like 5 (FcRL5)

The FcRL4 and FcRL5 were recently described as receptors for IgA and IgG, respectively (20). FcRL4 is expressed on B_TLM_ cells that lack the classical CD27 memory B cell marker, and FcRL4^+^ B cells localize at the subepithelial and marginal zones of mucosal lymphoid tissues. FcRL4^+^ B cells express switched immunoglobulins that have undergone variable region somatic mutations and secrete high levels of IgG but also IgA in response to stimulation with T cell cytokines, but not following B-cell receptor (BCR) cross-linking (21). FcRL5 is also expressed specifically on CD27 negative B cells, and FcRL5 cross-linking induces B cell proliferation and surface IgA and IgG expression, suggesting that FcRL5 participates in the expansion and development of antigen-primed B cells in physiological conditions (22). Thus, we determined the surface expression of these two receptors on penile B cells by flow cytometry. The frequency of FcRL4^+^ B cells was higher in the urethra (20.3% [16.8–23.9]) compared with the fossa (10.6% [8.2–12.9]) and glans (5.7% [3.1–8.2]) (Figure 2E, left panel). By contrast, the less abundant FcRL5^+^ B cells were equally present in all three regions [(1.3% [0.6–1.9]); (2.8% [1.8–3.7]); and (2.5% [1.3–3.7]) for urethra, fossa and glans, respectively] (Figure 2E, right panel).

### The Penis Contains Plasma Cells (PCs)

The capacity of penile B cells to secrete antibodies was inferred from the presence of CD38^+^CD138^+^ mucosal PCs among B cell populations in penile tissues. PCs were present in all penile regions representing less than 5% of total B cells in urethra (5% [4.5–5.6]), fossa (4.2% [3.3–5.0]), and glans (1.8% [0.9–2.6]) (Figure 2F). The frequency of PCs was higher in the urethra and fossa compared with glans.

Staining of the tissues for CD138/syndecan-1 expression followed by immunohistochemistry (Figure 2G) indicated that all penile regions contained CD138^+^ PCs in the lamina propria. In line with the flow cytometry results, the densities of PCs in the urethra and the fossa were greater than in the glans. The expression of syndecan-1 by keratinocytes precluded the specific detection of PCs in the penile epithelia using this marker.

Altogether, all three penile compartments, and especially the urethra, contain B cells of which small amounts are PCs. Penile B cells are mainly memory cells, with either a tissue-like memory or an activated memory phenotype. Expression of FcRL4 (and to a lesser extent FcRL5) and syndecan-1 together with their lamina propria localization are indicative of the presence of antibody-secreting PCs.

### Penile CD4^+^ and CD8^+^ T Cells Are Mainly Resting Effector Memory (EM) Cells

**Figure 3:**
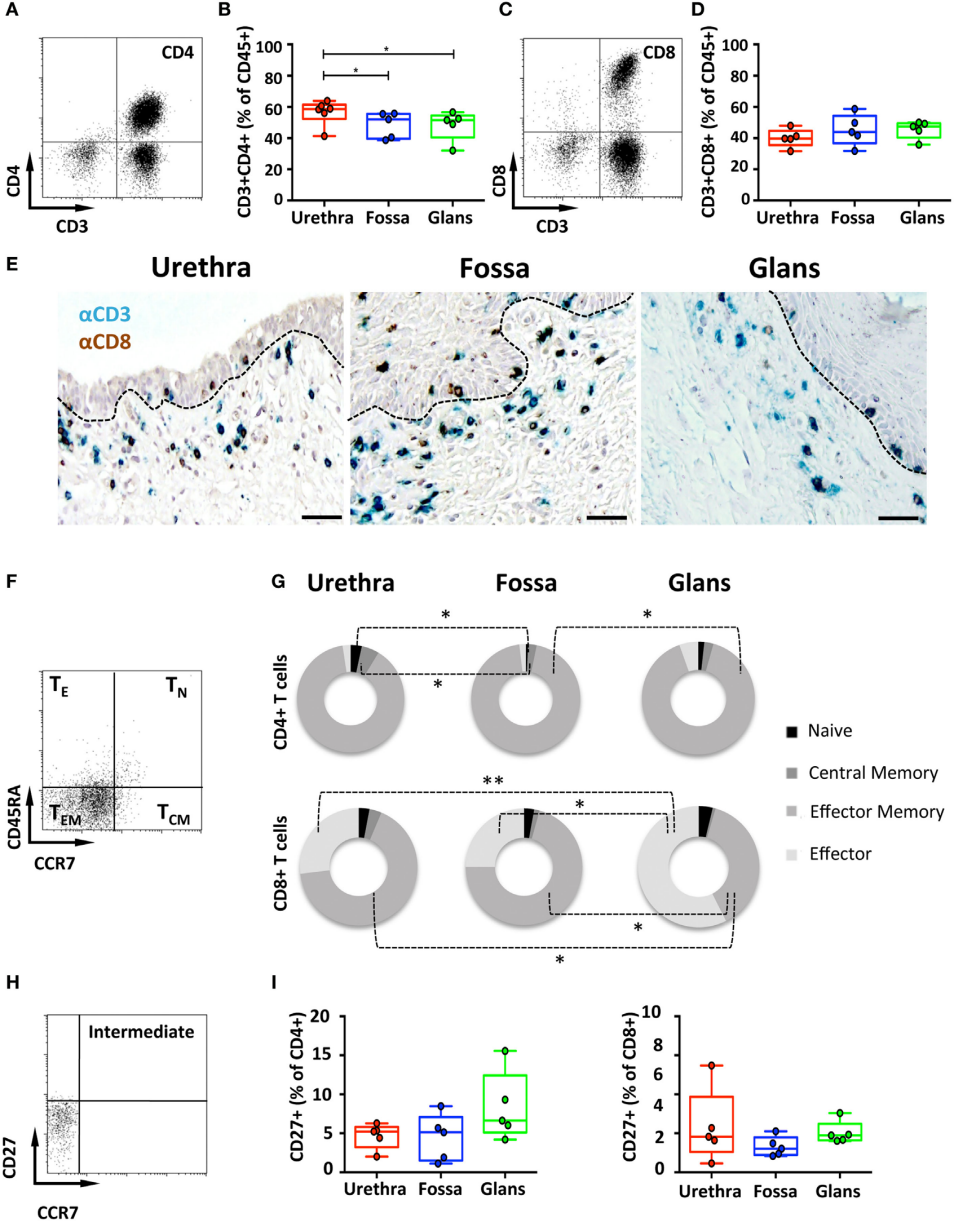
Characterization of penile CD4^+^ and CD8^+^ T cells. **(A,C)** Representative dot plots and **(B,D)** box-and-whisker plots of total CD45^+^CD3^+^CD4^+^ T **(A,B)** and CD45^+^CD3^+^CD8^+^ T **(C,D)** cells (referred to as CD4^+^ and CD8^+^ T cells, respectively) proportions in urethra, fossa, and glans [*n* = 6, 5, and 5 different donors for urethra, fossa, and glans, respectively; mean age 37 years (range: 22–56)]. **(E)** Immunohistochemistry analysis of CD4^+^ T (CD3^+^CD8^−^, blue) and CD8^+^ T (CD3^+^CD8^+^, black) cells in urethra, fossa, and glans [representative of *n* = 5 different donors for each tissue; mean age 41 years (26–54)]. Cells were stained with anti-CD3 and anti-CD8 antibodies and revealed using diaminobenzidine (brown staining) or histogreen (blue staining) peroxidase substrates. Scale bar = 50 μm. **(F)** Representative dot plot and **(G)** proportions of CD45RA^+^CCR7^+^ naive (T_N_), CD45RA^−^CCR7^+^ central memory (T_CM_), CD45RA^−^CCR7^−^ effector memory (T_EM_), and CD45RA^+^CCR7^−^ terminally differentiated effector (T_E_) CD4^+^ T (upper panel) and CD8^+^ T (lower panel) cells in penile tissues [*n* = 5 different donors for each tissue; mean age 37 years (22–5)]. **(H)** Representative dot plot and **(I)** proportions of intermediate memory CCR7^−^CD27^+^CD4^+^ T (left panel) and CD8^+^ T (right panel) cells in urethra, fossa, and glans [*n* = 5 different donors for each tissue; mean age 46 years (28–56)]. All box-and-whisker plots represent minimum-to-maximum values, and each point corresponds to one donor. Statistical analyses were performed first by the Kruskal–Wallis test; pairwise comparisons were performed by the Mann–Whitney *U*-test. *p < 0.05, **p < 0.01, and ***p < 0.001.

CD4^+^ and CD8^+^ T cells are key players in the adaptive immune response. To identify their presence and profile in penile tissues, the frequency of cells co-expressing CD3/CD4 and CD3/CD8 was determined amongst CD45^+^ population (Figures 3A,C, respectively). In the urethra, CD4^+^ T cells predominated over CD8^+^ T cells, representing 58.6% [55.5–61.8] and 39.7% [37.1–42.2] of CD3^+^ T cells, respectively. In fossa and glans, CD4^+^ T cells were present in equivalent proportions, and significantly lower than in the urethra (52% [48.4–55.6], *p* = 0.0219 and 51.5% [47.3–55.7], *p* = 0.0303, respectively). CD8^+^ T cells were equally present in all penile regions (see above for the urethra, 43.9% [39.5–48.2] and 47.5% [45–50] of CD3^+^ cells for fossa and glans, respectively) (Figures 3B,D). Tissue distribution of CD4^+^ and CD8^+^ T cells was also evaluated by immunohistochemistry following co-labeling with anti-CD3 (blue staining) and anti-CD8 (brown staining) antibodies. Hence, CD8^+^ T cells co-express CD3 and CD8 (blue/brown staining), whereas CD4^+^ T cells expressed CD3 but not CD8 (blue staining only) (Figure 3E). Matched isotypes served as negative control (data not shown). In the urethra, fossa, and glans, both CD4^+^ and CD8^+^ T cells localized in the lamina propria, close to the mucosal epithelium, whereas the majority of cells within the epithelial compartment were CD8^+^ T cells. However, it cannot be excluded that some of the cells detected by this technique also express T cell markers on their surface such as NKT cells.

Next, T cell maturation was assessed using CD45RA and CCR7 to distinguish four T cell subsets (23), namely, CD45RA^+^CCR7^+^ naive (T_N_), CD45RA^−^CCR7^+^ central memory (T_CM_), CD45RA^−^CCR7^−^ effector memory (T_EM_), and CD45RA^+^CCR7^−^ effector (T_E_) T cells. CD4^+^ T cells (Figures 3F,G, upper panel) mainly harbored a T_EM_ phenotype in all penile regions, consisting of 92.1% [89.8–94.4] in the urethra, 95.1% [93.5–96.7] in fossa, and 88.8% [84.6–92.9] in glans. CD4^+^ T_N_, CD4^+^ T_CM_, and CD4^+^ T_E_ represented each less than 5% of the total CD3^+^/CD4^+^ T cell population. Similar phenotypes applied to the CD3^+^/CD8^+^ T cells (Figure 3G, lower panel) that mainly consisted of T_EM_ with equivalent frequencies of 69.5% [63.4–75.7] and 70.0% [62.7–77.3], in the urethra and fossa, but significantly dropping to 35.5% [29.8–41.2] in the glans (*p* = 0.0159 vs urethra and fossa). T_E_ constituted an important glans CD8^+^ subset (53.5% [49.8–57.1] of CD8^+^ T cells), whereas in the urethra and fossa, CD8^+^ T_E_ dropped significantly (27.9% [22.1–33.9] and 24.8% [16.7–32.9], *p* = 0.0079 and 0.0156 vs the glans, respectively).

Intermediate memory T cells (T_IM_) were next quantified based on CD27 expression in the CCR7 negative memory T cells population (Figure 3H). T_IM_ represented around 5% of the memory population of both CD4^+^ and CD8^+^ T cells (Figure 3I), with 5.2% [4.5–5.9] and 4.5% [2.5–6.6] in the urethra, 5.1% [3.8–6.5] and 3% [2.5–3.6] in fossa, and 6.6% [4.7–8.6] and 4.7% [4.1–5.4] in glans.

**Figure 4:**
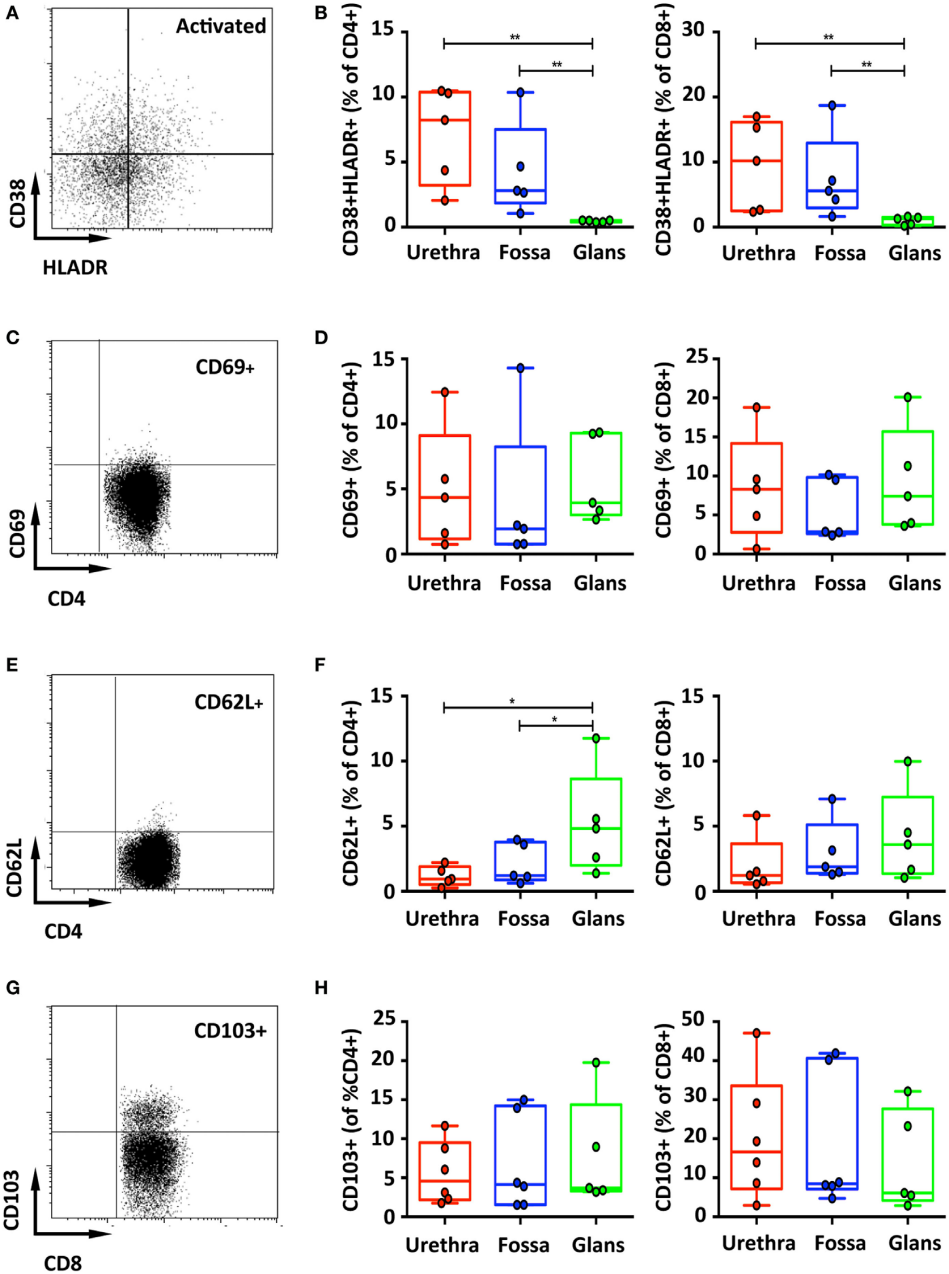
Activation and adhesion markers of penile T cells. Representative dot plots **(A,C)** and box-and-whisker plots of activated CD45^+^CD3^+^CD4^+^ (referred to as CD4+) [**(B,D)**, left panel] and CD45^+^CD3^+^CD8^+^ (referred to as CD8^+^) T cells [**(B,D)**, right panel]. HLA-DR^+^CD38^+^ **(A,B)** and CD69^+^ **(C,D)** activated T cells were detected in penile tissues by flow cytometry on CD4^+^ (left panel) and CD8^+^ (right panel) T cell populations [*n* = 5 different donors for each tissue; mean age 37 years (range: 22–56) for HLA-DR/CD38 staining and 46 years (28–56) for CD69 staining]. Representative dot plots and box-and-whisker plots representing expression of CD62L [**(E,F)**, respectively, *n* = 5 different donors for each tissue and mean age 46 years (28–56)] and the adhesion molecule CD103 [**(G,H)**, respectively, *n* = 6, 6, and 5 different donors for urethra, fossa, and glans, respectively. Mean age 40 years (23–51)] detected by flow cytometry on CD4^+^ (left panel) and CD8^+^ (right panel) T cell populations. All box-and-whisker plots represent minimum-to-maximum values, and each point corresponds to one donor. Statistical analyses were performed first by the Kruskal–Wallis test; pairwise comparisons were performed by the Mann–Whitney *U*-test. *p < 0.05, **p < 0.01, and ***p < 0.001.

Last, T cell activation, defined by co-expression of HLA-DR and CD38, was assessed (Figure 4A). In the urethra and fossa, CD4^+^ T cells exhibited a resting phenotype, as activated cells co-expressing CD38 and HLA-DR represented only 8.2% [6.7–9.7] and 2.8% [1.2–4.4] (Figure 4B, left panel). In the glans, activated cells were significantly lower and practically absent, representing only 0.5% [0.47–0.58] (*p* = 0.008 vs urethra and fossa). Penile CD8^+^ T cells, as CD4^+^ T cells, had also a resting phenotype (Figure 4B, right panel, 10.2% [7.2–13.2]; 5.6% [2.7–8.5]; 1.3% [1.0–1.6] in the urethra, fossa, and glans, respectively. *p* = 0.0079 for urethra vs glans and fossa vs glans).

### Resident Memory T Cells Form a Minor T Cell Subset in All Penile Regions

Memory T cells, particularly CD8^+^ cells that can remain in tissues without recirculation through the blood (24), are referred to as resident memory cells, and can be characterized by the expression of CD69, L-selectin (CD62L) and CD103 (αEβ7 integrin). Both CD4^+^ and CD8^+^ T cells poorly expressed CD69 (Figures 4C,D) and CD62L (Figures 4E,F). By contrast, T cells expressing CD103 dominated in the CD8^+^ subset, with 16.7% [10.3–23.1] in the urethra, 8.5% [1.5–15.5] in the fossa, and 6.1% [0.4–11.8] in the glans, compared with only around 5% expression in the CD4^+^ subset in all penile regions (4.6% [3–6.2]; 4.1% [1.7–6.6]; 3.7% [0.6–6.8] in the urethra, fossa, and glans, respectively) (Figures 4G,H), although their distribution was very heterogeneous between individuals.

Overall, CD4^+^ and CD8^+^ T cells are equally present in all penile tissue regions and harbor a resting phenotype. Whereas CD4^+^ T cells are mostly (>90%) EM cells, CD8^+^ T cell subsets include mainly EM cells but also effector cells, especially in the glans. Furthermore, the higher expression of CD103 by CD8^+^ relative to CD4^+^ T cells translates into a higher epithelial CD8^+^ T cells distribution than that of CD4^+^ T cells, although both subsets are equally present in the lamina propria.

### Cytokines Produced by Penile Cells

T helper (Th) cells represent one of the most important cell type in adaptive immunity, as they are required for almost all adaptive immune responses. Once activated, Th cells secrete different types of cytokines that in turn stimulate other immune cells such as B cells, which provide help to secrete antibodies or cytotoxic T cells for killing infected target cells, thereby amplifying the immune response against pathogens (25). Flow cytometry was used to determine the frequency of cells producing Th1-like cytokines, namely, IFN-γ, TNF-α, and IL-2, cells producing Th2-like cytokines as IL-4 and IL-5 and cells producing IL-17 and IL-22 in the different penile mucosa.

**Figure 5:**
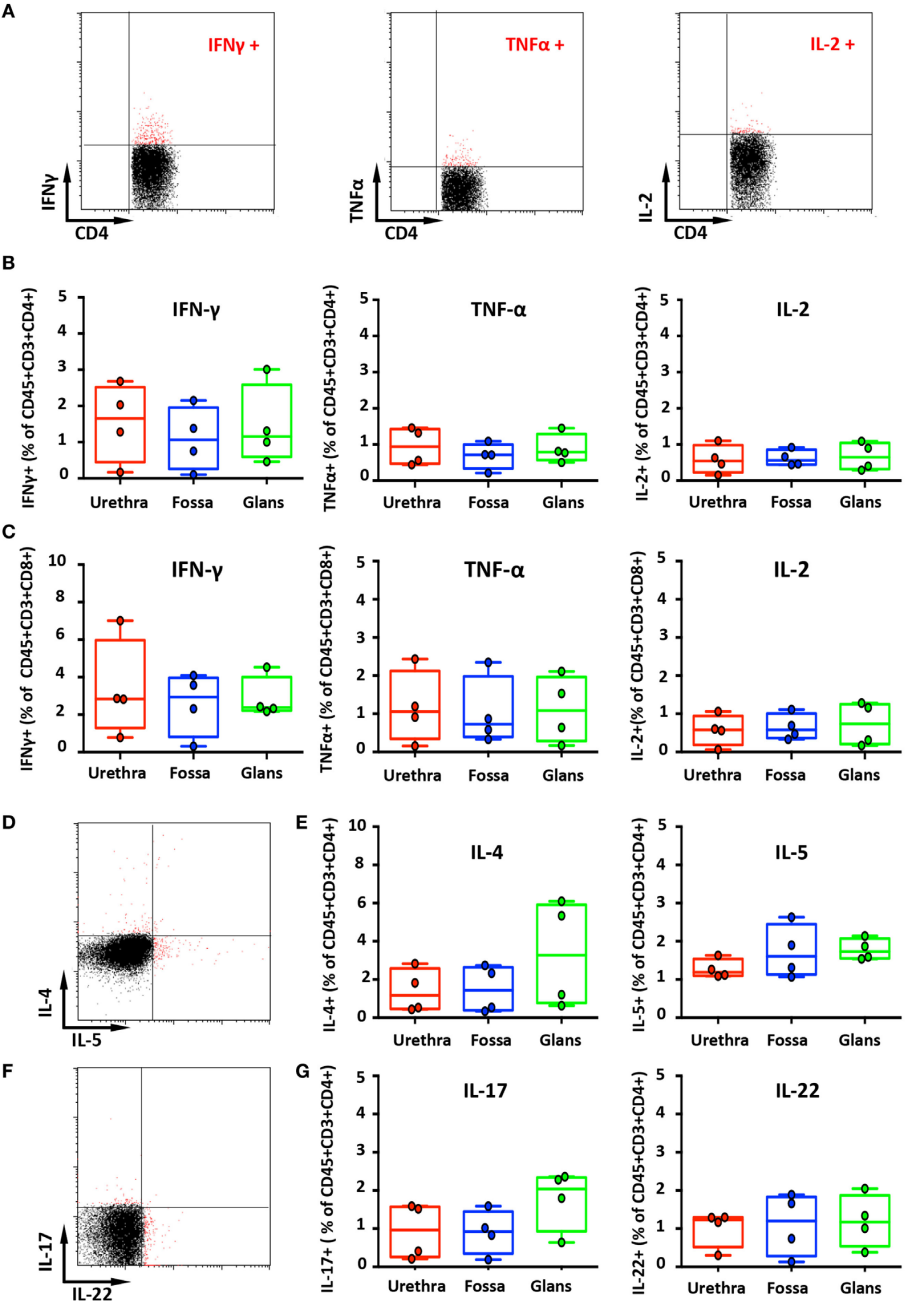
Secretion of cytokines by penile cells. **(A)** Representative dot plots of spontaneous secretion of IFN-γ (left panel), TNF-α (middle panel), and IL-2 (right panel) by urethral CD45^+^CD3^+^CD4^+^. **(B,C)** Box-and-whisker plots representing the spontaneous secretion of IFN-γ (left panel), TNF-α (middle panel), and IL-2 (right panel) by penile CD45^+^CD3^+^CD4^+^ **(B)** and CD45^+^CD3^+^CD8^+^ cells **(C)** after intracellular staining in the different penile tissues [*n* = 4 different donors for each tissues; mean age 34 years (range: 25–51)]. Representative dot plots **(D)** and box-and-whisker plots **(E)** representing the spontaneous secretion of IL-4 [**(E)**, left panel] and IL-5 [**(E)**, right panel] by penile CD45^+^CD3^+^CD4^+^ cells after intracellular staining in urethra, fossa, and glans [*n* = 4 different donors for each tissues; mean age 34 years (25–51)]. Representative dot plots **(F)** and box-and-whisker plots **(G)** representing the spontaneous secretion of IL-17 [**(G)**, left panel] and IL-22 [**(G)**, right panel] by penile CD45^+^CD3^+^CD4^+^ cells after intracellular staining in the different penile tissues [*n* = 4 different donors for each tissues; mean age 34 years (25–51)]. All box-and-whisker plots represent minimum-to-maximum values, and each point corresponds to one donor.

IFN-γ-secreting cells were detected in all penile tissues, with a predominance for CD8^+^ cells (2.8% [1.6–4.1], 2.9% [1.9–4.0], and 2.4% [2.3–2.4] in the urethra, fossa, and glans, respectively) (Figure 5C, left panel) by comparison with CD4^+^ cells (1.7% [1.1–2.2], 0.8% [0.4–1.1], and 1.2% [0.9–1.4] in the urethra, fossa, and glans, respectively) (Figures 5A,B, left panel). However, TNF-α (Figures 5B,C middle panels) and IL-2 (Figures 5B,C, right panels) secreting cells were low in these tissues (less than 1% of CD4^+^ and CD8^+^ cell populations, representative dot plots for CD4^+^ cells shown on Figure 5A).

The secretion of Th2-like cytokines by CD4^+^ cells was also evaluated (Figures 5D,E). CD4^+^ cells expressing IL-4 were detected in equivalent proportions in the urethra and the fossa (1.2% [0.6–1.7] and 1.4% [0.9–1.9], respectively) and slightly more abundant in the glans (3.3% [2.2–4.3]) (Figure 5E, left panel). On the other hand, CD4^+^ cells secreting IL-5 were in equal proportions in the urethra (1.2% [1.1–1.3]), the fossa (1.6% [1.4–1.8]), and the glans (1.7% [1.6–1.8]) (Figure 5E, right panel).

Th17 cells correspond to a pro-inflammatory subset of CD4^+^ T cells that secrete IL-17 after stimulation. Th17 cells provide a protective inflammatory response toward pathogens in mucosal tissues, such as skin, gut, and lung. IL-17 mediates the recruitment of neutrophils and macrophages to infected tissues, thereby acting in host defense against extracellular pathogens (26). Although CD3^+^CD4^+^ cells from all penile tissues secreted IL-17 (Figures 5F,G, left panel), IL-17 production in the glans appeared to be slightly higher (2.0% [1.7–2.3]), compared with the urethra and fossa (1% [0.6–1.3] and 0.9% [0.7–1.2], respectively). Finally, we evaluated the secretion by penile CD4^+^ cells of IL-22, a cytokine that modulates the expression of many genes encoding proteins involved in tissue protection, survival, differentiation, and remodeling, possibly exerting pro-inflammatory functions (26) (Figures 5F,G, right panel). Cells secreting IL-22 were equally frequent in the urethra (1.2% [1–1.4]), the fossa (1.2% [0.9–1.5]), and the glans (1.2% [1–1.3]).

### Penile NK Cells Have an Activated Profile and the Machinery to Mediate Antibody-Dependent Cell Cytotoxicity (ADCC)

Natural killer cells, phenotypically defined as CD3^−^CD56^+^ lymphocytes, are part of the innate immune system acting as first line of defense of the organism due to their capacity of destroying infected cells without prior activation/stimulation. The intensity of CD56 expression distinguishes two subpopulations of NK cells (27), namely, the CD56^dim^ cytotoxic and the CD56^bright^ immunoregulatory NK cells.

**Figure 6:**
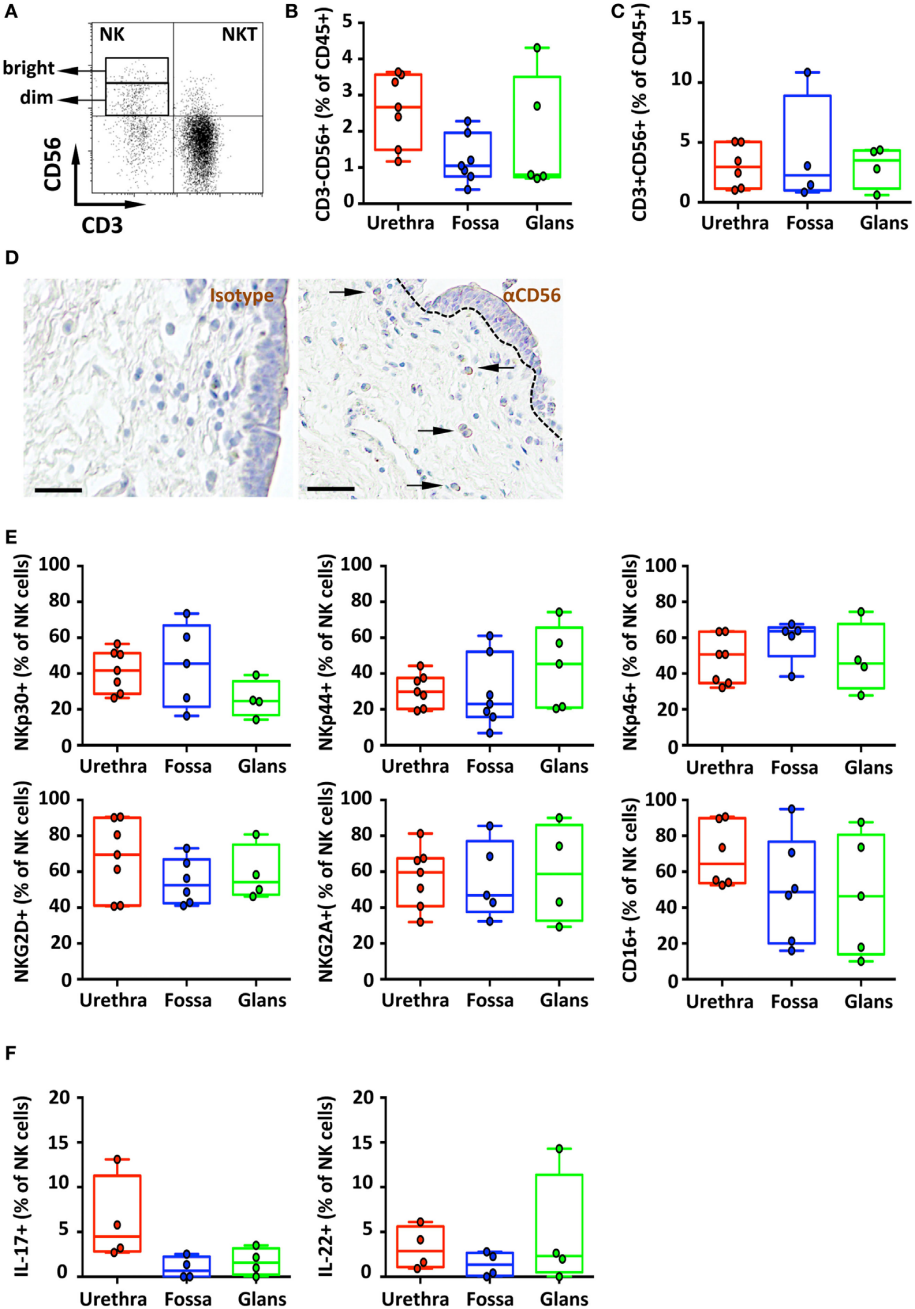
Phenotype and distribution of penile natural killer (NK) and NKT cells. Representative dot plot **(A)** and box-and-whisker plots of **(B)** total CD45^+^CD3^−^CD56^+^ NK cells [*n* = 8, 7, and 5 different donors for urethra, fossa, and glans, respectively; mean age 35 years (range: 19–56)] and **(C)** total CD45^+^CD3^+^CD56^+^ NKT cells detected by flow cytometry in urethra, fossa, and glans [*n* = 6, 4, and 4 different donors for urethra, fossa, and glans, respectively; mean age 35 years (19–56)]. **(D)** Immunohistochemistry analysis of CD56^+^ cells (right panel) by comparison with a mouse IgG1 isotype control (left panel) in urethral tissues [representative of *n* = 5 different donors; mean age 41 years (26–54)]. Cells were stained with an anti-CD56 antibody or a mouse IgG1 isotype control and revealed using diaminobenzidine peroxidase substrate (brown staining). Scale bar = 50 μm. **(E)** Expression pattern of NKp30, NKp44, NKp46, NKG2D, NKG2A, and CD16 receptors on CD45^+^CD3^−^CD56^+^ NK cells in penile mucosae [*n* = 4–7 different donors; mean age 35 years (19–56)]. **(F)** Box-and-whisker plots representing the spontaneous secretion of IL-17 (left panel) and IL-22 (right panel) by penile CD45^+^CD3^−^CD56^+^ NK cells after intracellular staining in urethra, fossa, and glans [*n* = 4 different donors for each tissue; mean age 34 years (25–51)]. All box-and-whisker plots represent minimum-to-maximum values, and each point corresponds to one donor.

CD45^+^CD3^−^CD56^+^ NK cells (Figure 6A) in the urethra, fossa, and glans constituted less than 3% of total CD45^+^ leukocytes (2.7% [2.3–3.0]; 1% [0.8–1.3]; and 0.8% [0.1–1.5], respectively) (Figure 6B) and were mainly CD56^dim^ (Figure 6A). We can also detect CD45^+^CD3^+^CD56^+^ cells (Figures 6A,C) in these tissues, which correspond to NKT cells. This population represents 3.0% [2.2–3.7], 2.2% [0–4.5], and 3.5% [2.6–4.4] of total CD45^+^ penile cells in the urethra, fossa, and glans, respectively.

In line with this CD56^dim^ NK cell profile, CD56^+^ cells were detected at low, but specific, frequency in the lamina propria of the urethra after comparative immunochemical detection with an anti-CD56 and isotype control-matched antibody (Figure 6D, right and left panels, respectively). However, CD56^+^ cells appeared absent from the fossa and the glans using this technique (data not shown), most likely because CD56 expression was too low for visualization by immunohistochemistry.

The main activating receptors of penile NK cells, namely, the members of the natural cytotoxicity receptors (NCR) family NKp30, NKp44, and NKp46 (27), were expressed in different proportions in the urethra, fossa and glans (Figure 6E, upper panel). NKp44, an exclusive marker of activated cells (23), was expressed on NK cells from the urethra (29.9% [26.5–33.3]), fossa (23% [15.7–30.4]), and glans (45.3% [35.2–55.5]) (Figure 6E, upper panel, middle). In addition, NK cells in all penile regions also expressed NKp30 and NKp46 (Figure 6E, upper panel, right and left) as well as the activating receptor NKG2D and the inhibiting receptor NKG2A (Figure 6E, lower panel). Finally, the FcγRIII (CD16) was also highly expressed by NK cells (Figure 6E, lower panel, right) from the urethra (64.4% [57.2–71.5]), fossa (47.7% [35.8–59.6]), and glans (46.4% [31.7–61.1]), suggesting that penile NK cells can bind IgGs thus acting as effector cells to mediate ADCC in pathologic conditions (Figure 6E, bottom panel).

In addition to T cells, innate immune cells including NK cells also secrete the inflammatory cytokines IL-17 and IL-22 (28). We thus evaluated whether IL-17 was spontaneously produced by penile NK cells. Only urethral NK cells secreted IL-17 (4.5% [3.6–5.4]) unlike those from fossa and glans (0.7% [0–1.5] and 1.6% [1–2.2], respectively) (Figure 6F, left panel). By contrast, NK cells from all penile compartments produced IL-22 (2.9% [1.9–3.8], 1.3% [0.5–2.2], and 2.3% [0–6.7] in the urethra, fossa, and glans, respectively) (Figure 6F, right panel).

Altogether, all penile tissue regions are equally populated by NKp44^+^-activated NK cells residing in the lamina propria that are equipped to trigger ADCC *via* the CD16 receptor, and are able to secrete spontaneously pro-inflammatory cytokines such as IL-17 and IL-22.

### Homing Receptors Expression by Penile Immune Cells

**Figure 7:**
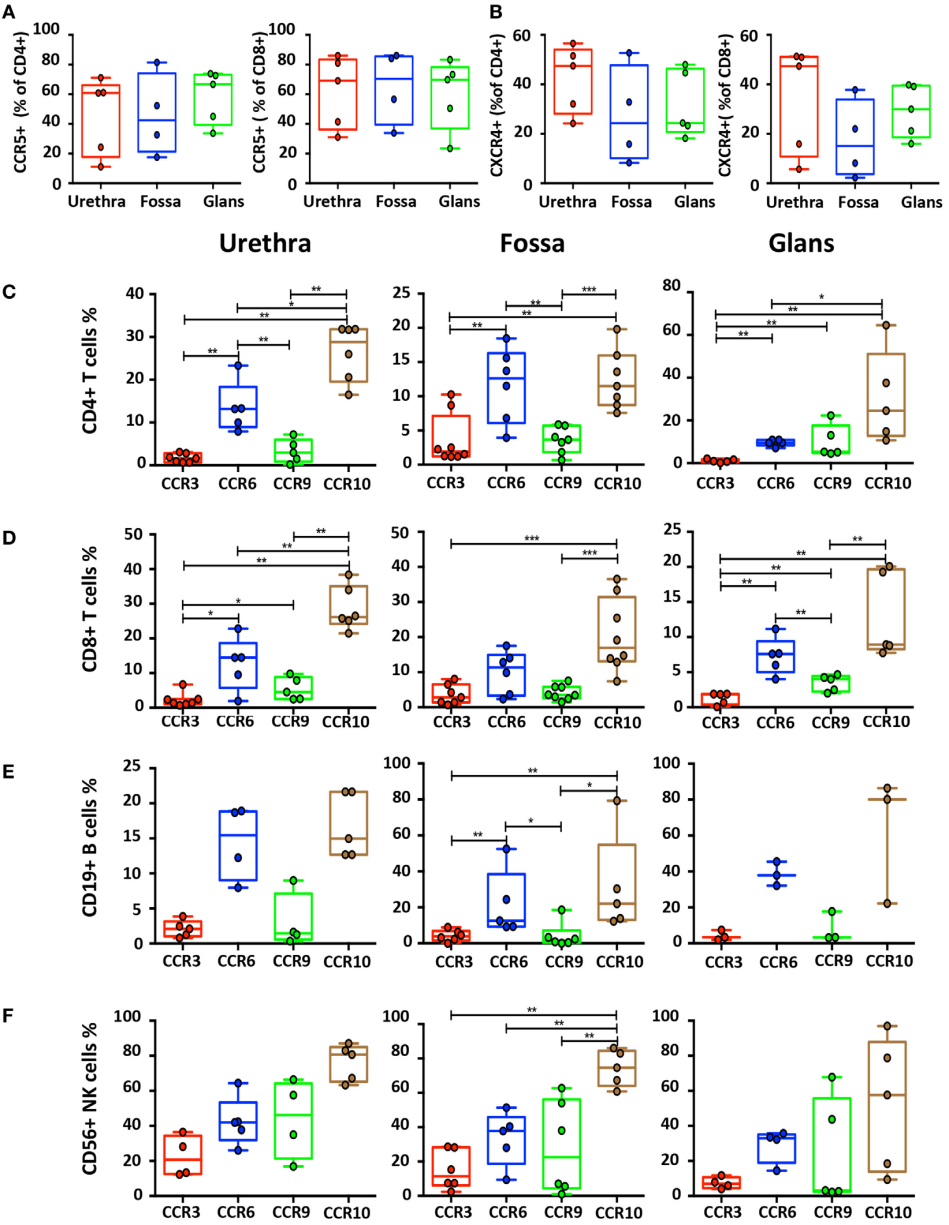
Characterization of homing receptors profiles on the different penile cell populations. Box-and-whisker plots of CD45^+^CD3^+^CD4^+^ and CD45^+^CD3^+^CD8^+^ T cells expressing **(A)** CCR5 and **(B)** CXCR4 receptors in urethra, fossa, and glans [*n* = 5, 4, and 5 different donors, respectively; mean age 31 years (range: 22–43)]. **(C–F)** Box-and-whisker plots representing the expression of the CCR3, CCR6, CCR9, and CCR10 homing receptors on CD45^+^CD3^+^CD4^+^ T cells **(C)**, CD45^+^CD3^+^CD8^+^ T cells **(D)**, CD45^+^CD3^−^CD19^+^ B cells **(E)**, and CD45^+^CD3^−^CD56^+^ natural killer (NK) cells **(F)** in urethra (left panel), fossa (middle panel), and glans (right panel) detected by flow cytometry [*n* = 3–8 different donors; mean age 37 years (19–58)]. All box-and-whisker plots represent minimum-to-maximum values, and each point corresponds to one donor. Statistical analyses were performed first by the Kruskal–Wallis test; pairwise comparisons were performed by the Mann–Whitney *U*-test. *p < 0.05, **p < 0.01, and ***p < 0.001.

We first evaluated CCR5 and CXCR4 expression on T cells (Figures 7A,B), two chemokine receptors that additionally permit HIV-1 infection. CCR5^+^CD8^+^ T cells were more numerous than CCR5^+^CD4^+^ T cells in the urethra (69.2% [58.5–79.8] vs 60.9% [49.4–72.4]), fossa (70.4% [58.2–82.5] vs 42.5% [28.9–55.9]), and glans (69.8% [59.4–80.1] vs 66.7% [58.8–74.6]). Furthermore, both CD4^+^ and CD8^+^ T cells expressed higher level of CXCR4 in the urethra (47.5% [41.5–53.4] and 47.4% [37.8–56.9], respectively) compared with CD4^+^ and CD8^+^ T cells from fossa (24.3% [13.2–35.5] and 15.1% [7.3–22.8], respectively) and glans (24.4% [18.4–30.3] and 30% [25.3–34.6], respectively).

Next, we assessed the surface expression of homing receptors, namely, CCR3, CCR6, CCR9, and CCR10. CCR6 and CCR10 were expressed at a significantly higher level than CCR3 and CCR9 on B, CD4^+^, and CD8^+^ T cells in all penile regions (Figures 7C–E). Furthermore, expression of CCR6 and CCR10 on NK cells (Figure 7F) in the different penile regions was higher compared with B, CD4^+^, and CD8^+^ T cells. Finally, whereas less than 5% of B and T cells expressed CCR3 and CCR9 in all penile regions (Figures 7C–E), NK cells expressed higher levels of both CCR3 in the urethra, and CCR9 in all penile compartments (Figure 7F).

Thus, CCR10 and CCR6, and to a lesser extend CCR3 and CCR9, might participate in the differential homing of these mucosal immune cells into penile tissues.

### Urethra, Fossa, and Glans Have Different Cytokine Environments

**Figure 8:**
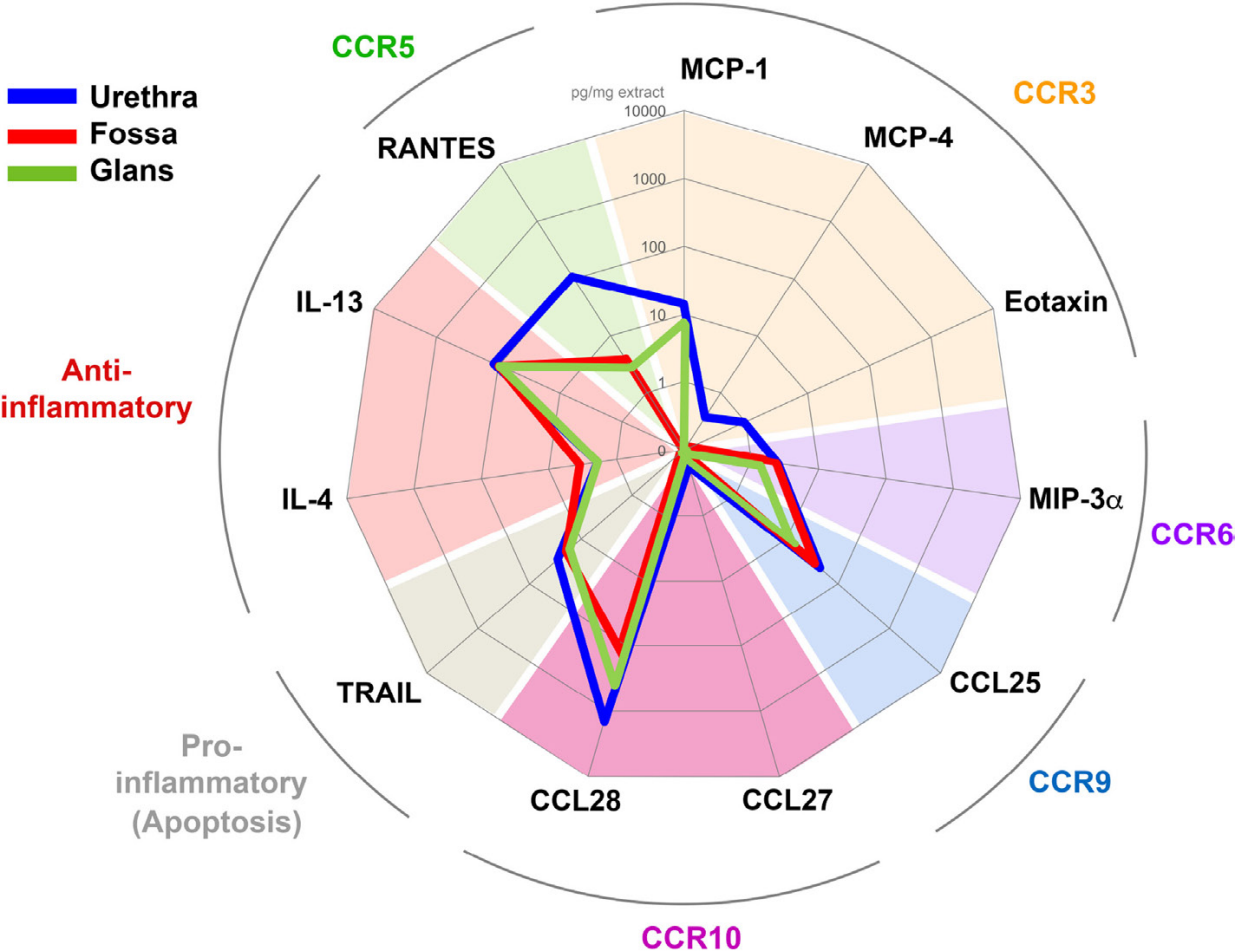
Penile tissues have different cytokine environments. Radar representation of the different mean concentrations of cytokines/chemokines detected in urethra (blue line, *n* = 6), fossa (red line, *n* = 3), and glans (green line, *n* = 3). Each concentration is expressed in picogram per milligrams of protein tissue extract and is represented on a logarithmic scale. Each cytokine/chemokine is grouped according to its specificity for their respective homing receptor or their immunological function as indicated in colored characters outside the circle.

Cell homing is driven by chemokine receptor expression, and their capacity to bind to the appropriate chemokines/cytokines expressed specifically in each target mucosal tissue. We therefore evaluated which chemokines/cytokines were constitutively expressed in the various penile mucosal regions. Tissue pieces derived from the three penile regions were lysed, and the supernatants were analyzed for the presence of MCP-1/CCL2, MCP-4/CCL13, Eotaxin/CCL11 (ligands of CCR3), MIP-3α/CCL20 (one ligand of CCR6), CCL25 (the ligand of CCR9), CTACK/CCL27 and CCL28 (the ligands of CCR10), RANTES/CCL5 (one ligand of CCR5), TRAIL/TNFS10 (a member of the tumor necrosis factor family of ligands capable of initiating apoptosis through engagement of cell death receptors), and the inflammatory cytokines IL-4 and IL-13 using the luminex and quantikine technologies (Figure 8).

In the urethra (Figure 8, blue line) CCL27, MCP-4, Eotaxin, MIP-3α, and IL-4 were detected at low concentrations (0.2–1.9 pg/ml mg of tissue, *n* = 6 different tissues), whereas RANTES, IL-13, and CCL28 concentrations were 100 times higher (108.0–1,436.5 pg/ml mg of tissue, *n* = 6 different tissues). MCP-1, TRAIL, and CCL25 concentrations were intermediate (14.0–44.1 pg/ml mg). In fossa and glans (Figure 8, red and green lines, respectively), MIP-3α, IL-4, IL-13, TRAIL, and CCL25 were detected at concentrations equivalent to those measured in the urethra. However, CCL28 and RANTES concentrations were 10 and 100 times lower than that in the urethra, respectively. Finally, MCP-1 was detected in the glans and urethra but not in the fossa.

Altogether, the tissue cytokine milieu varies with the penile tissue regions, with the urethra appearing to be the richest one.

## Discussion

Numerous pathogens including viruses and bacteria use the human penis as a portal of entry into the body and a vaccine locally active against such pathogens is key to control the spread of STIs. Establishment of immune memory against pathogens occurs naturally after infection and permits to establish a faster specific immune response following a second contact with the pathogen.

Unlike that of the other penile regions, the mucosal immune system of the foreskin has been intensively studied and characterized. Thus, the lamina propria and the epithelium of the human foreskin selectively contain macrophages and CD1a^+^HLA-DR^+^ LCs, respectively (6, 7, 9). Furthermore, the foreskin also harbors dendritic cells and numerous EM T cells, including CD4^+^ Th17 cells (29). Finally, the foreskin epithelium is also protect by Mucin proteins, such as MUC1 and MUC4, and expresses TLRs, particularly TLR5 (16), TLR4 and TLR3 (30), thus endowing this tissue with an important anti-infectious potential.

An effective vaccine must ideally induce such pathogen-specific immune memory by inducing local specific humoral and cellular responses, in turn preventing mucosal pathogen entry and establishment of infection. Therefore, the first requirement for elaborating a protective vaccine in the penis is to evaluate whether the penile tissue possesses the appropriate set of mucosal immune cells able to target each penile compartment, and to develop a protective local immune response.

Mucosal B cells, and particularly the most abundant memory B cells we found in all penile regions, are fundamental for the development of a long-lived vaccine humoral response. Long-lived humoral memory comprises two major components, namely, memory B cells that search for their specific antigen in the periphery allowing for later affinity maturation, proliferation, and differentiation (31), and the PCs that locally produce specific immunoglobulins.

Penile memory B cells comprise CD21^−^CD27^−^FCRL4^+^ B_TLM_, most abundant in all penile regions and reaching even 75% of glans B cells. B_TLM_ that lack the memory marker CD27 are an exhausted B cells subset (32), which also express numerous inhibitory receptors containing immunoreceptor tyrosine-based inhibitory motifs as well as FcRL4 (32). The high expression of FcRL4 on urethral B cells we report may control BCR-dependent proliferation (19, 31, 33). Hence in the context of HIV-1 infection, FcRL4 in B_TLM_ cells negatively controls the production of cytokines by B_TLM_ cells, but also limits the secretion rate of HIV-1 specific antibodies.

Penile memory B cells include also the CD21^−^CD27^+^ B_AM_ that are long-lived, express the BCR, and have already undergone the processes of class switching and antibody affinity maturation. Upon pathogen infection, these B_AM_ transform into CD27^+^CD38^+^CD138^+^ PCs that will produce large amounts of antibodies, particularly IgG or IgA. B_AM_ represent 30% of total B lymphocytes in the urethra and fossa but only 15% in the glans. CD38^+^CD138^+^ PCs were found in all penile compartments although in limited quantity, residing in the tissue lamina propria. Although we did not determine the immunoglobulin isotype secreted by penile PCs, these cells in the urethra and fossa are known to predominantly secrete polymeric IgA and IgM that translocate through urethral epithelium in a polymeric immunoglobulin receptor-dependent manner, thus releasing secretory SIgA or SIgM in the urethral canal (5, 6). In addition, mucosal PCs secreting IgG locally are also present in various mucosa (34) including the foreskin (35) where IgGs translocate across the epithelium in a FcRn-dependent manner (36). Thus B_AM_ cells and in turn PCs will most likely respond locally to a mucosal vaccine, allowing a rapid and specific response against the targeted pathogen.

How B cells home specifically into the various penile compartments remains unclear. Among the chemokine receptors expressed on penile B cells in the urethra, fossa, and glans, we now show that CCR6 and CCR10 likely drive penile homing, although CCR6 usually mediates mucosal homing to inflamed tissues including lung and gut (37). Especially in the urethra, CCR6 might promote homing as CCR6 chemotaxes toward CCL20/MIP-3α, a chemokine we found enriched in the urethral compartment. Furthermore CCL28, one ligand of CCR10 detected in all penile tissues, might also participate in penile homing of B cells, as in the female genital tract (38).

In our study, CD3^+^ T cells represent the major immune cell population of all the penile regions. Although CD8^+^ T cell-mediated immunity is crucial in antiviral defense, CD4^+^ T cells remain the determinant T cell subpopulation mediating a wide variety of actions on other immune actor cells, either directly by cell–cell contact or indirectly *via* chemokine/cytokine secretion. Hence, CD4^+^ T cells represent half of all T cells in all penile regions, thus contrasting with the female genital tract (39). Of note, using morphological studies, other reported that CD8^+^ predominate over CD4^+^ T cells in the male genital tract (6), although this apparent contradiction most likely resides in the differences in techniques used, namely, immunohistochemistry (6) vs the more quantitative flow cytometry approach we used here.

Similar to other genital tissues, such as the foreskin (29), cervix and endometrium (40), and other mucosa (41), we show here that resting EM cells are the dominant T cell subset in all penile compartments. Within the CD4^+^ T population, EM cells are long-lived cells derived from effector cells, which migrate into infected tissues to eliminate. Upon reinfection, CD4^+^ T_EM_ secrete different effector cytokines and chemokines resulting in a faster and more efficient secondary response.

All penile tissues contained cells able to secrete pro- and anti-inflammatory cytokines that regulate the immune response and the recruitment of immune cells to sites of pathogen infection (25). Their proportions in the different penile mucosa correspond to that described in the foreskin (29) or the sigmoid colon (42, 43), but differ from that of the female genital tract mucosa such as the endometrium (44). As penile T cells are resting cells, one might suggest that upon activation, as can occur during infection, for example, by HIV, these T cell subsets are induced to secrete their characteristic cytokines.

Of note, the amount of effector T cells among the CD8^+^ T cell population is higher than that of CD4^+^ T cell population, especially in the glans. Unlike their CD4^+^ counterparts, CD8^+^ effector cells correspond to a terminally differentiated cell population and are derived from T_EM_ (45). Thus, the increased presence of these effector cells in the lamina propria of the different penile tissues reflects an intense immune response, but also a continuous immunological monitoring at these sites.

Penile CD8^+^ T cells are essentially memory cells that express the mucosal αE/β7 integrin (CD103) which could explain their epithelial localization throughout the penile regions. Accordingly, this homing receptor retains T cells in genital epithelia but also in other mucosa, such as the gut, by interacting with E-cadherin expressed by epithelial cells (46). Such CD103^+^ T cells are most likely the non-recirculating resident memory T cell subset known to remain in tissues and prone to initiate a rapid immune response after resensitization (24).

Conversely, the recirculating T cell subset looses expression of CD103 and also the early activation marker CD69, thus cells devoid of these two markers might represent penile CD4^+^ T cells. Indeed, CD3^+^CD4^+^CD103^−^CD69^−^ cells are present in all penile regions studied here, similar to most non-lymphoid tissues (40). This particular CD103^−^CD69^−^ double negative phenotype associated with the expression of the HIV-1 co-receptor CCR5 on penile CD4^+^ T cells might explain the dissemination of HIV-1 in the host following penile infection of resident urethral macrophages (10), and potential subsequent transfer to CD4^+^ T cells. Although CD103 participates in the mucosal homing and residence of T cells in the female genital tract (46), the role of other receptors implicated in T cells homing into male or female genital tissues remains elusive, although T cells homing in other mucosa is better documented. For example, CCR9 and the α4β7 integrin participate in homing to the gut (47), whereas CCR3 and CCR10 are involved in T cell-mediated skin inflammation (48) and in CD4^+^ T-cell infiltration in the nasal mucosa (49). We suggest here that in addition to CCR10 and CCR6, CCR5 that is also expressed by penile T cells might participate in penile tissue homing due to the high concentration, especially in the urethra and fossa, of penile RANTES/CCL5, the natural ligand of CCR5.

Innate immune cells are also present in penile tissues including macrophages, as we have shown earlier (10), and NK cells as we reported here, similar to other genital tissues. Hence, vaginal NK cells are among the first ones recruited to sites of new infections, along with other innate and adaptive immune cells (50). Furthermore, vaginal NK cells participate in the control of pathogens such as HSV, as patients with defective NK cells show an increased susceptibility to HSV infections (50). Throughout the penis, NK cells are essentially CD56^dim^ and harbor the Fc-γ Receptor CD16, associated with the “cytotoxic” NK cell subset described in the blood. Such CD56^dim^ NK cells proliferate little and produce low level of cytokines, at least *in vitro* (27). Accordingly, CD16 is the principal Fc-γ receptor of glans NK cells (51). Furthermore, penile NK cells strongly express all the members of the NCR family (27), namely, NKp30, NKp44, and NKp46 as we show herein. NKp30 and NKp46 are constitutively expressed on all NK cells of healthy individuals, whereas NKp44 is expressed only after activation (23, 27). Penile NK cells are phenotypically similar to lung NK cells that are also CD56^dim^CD16^+^ and mostly NKp46^+^ (52), thus corresponding to blood NK cells. By contrast, penile NK cells differ from CD56_bright_CD16_+/–_ NK cells gut (53) and female reproductive tract (FRT) (54) that are comparable to immature NK cells found in secondary lymphoid tissues and known to produce higher amounts of inflammatory cytokines and harbor low cytotoxicity potential.

NKp44^+^ and NKp46^+^ expression indicates that penile NK contains the two subsets of NK cells identified at the mucosal level, namely, the NKp44^+^ NK cells able to secrete IL-17 and IL-22, and the NKp46^+^ NK cells producing IFN-γ (28, 55). IL-17 is a pro-inflammatory cytokine allowing for the accumulation of immune cells on the mucous surface, but also for the secretion of antimicrobial peptides by epithelial cells (26), whereas IL-22 confers protection to mucosal surfaces against bacterial and fungal infection, and promotes inflammation and the recruitment of immune cells at these sites (43). In addition, one of the other roles of IL-22 is to stimulate the secretion of the anti-inflammatory cytokine IL-10, by the epithelial cell barrier, allowing the preservation of its integrity (56). The secretion of these cytokines depends on IL-23 that is mainly produced by DCs, macrophages, and NK cells themselves following stimulation by lipopolysaccharides and the activation of the TLR4 as part of the innate immunity.

Thereby, in addition to their immune surveillance and putative cytotoxic functions, penile NK cells are therefore involved in the inflammatory reactions, thus allowing the establishment of the immune response orchestrated by B and T cells previously described.

**Figure 9:**
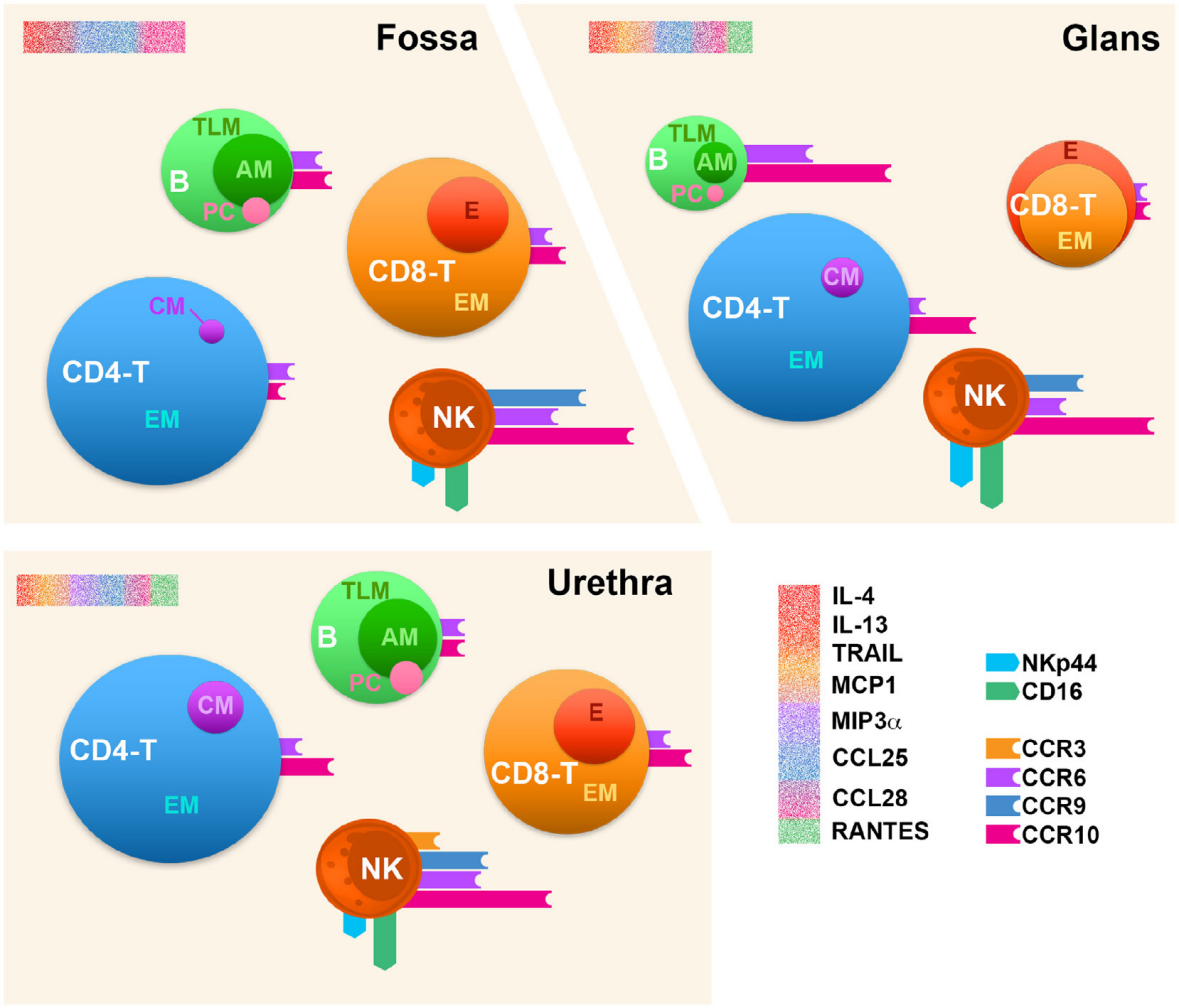
Immune cell distribution in the human penile tissue compartments. Penile CD4^+^/CD8^+^ T cells (CD4^−^ T and CD8^−^ T), B cells (B), and natural killer (NK) cells’ profiles in the three penile compartments, namely, urethra, fossa, and glans tissues based on the comparison of median values per each analysis. The size of each sphere corresponds to the percentage of cells relative to CD45^+^ leukocytes. CM, central memory; EM, effector memory; E, effector; TLM, tissue-like memory; AM, activated memory; PC, plasma cell. The expression of homing receptors (CCR3, CCR9, CCR6, and CCR10), the chemokine receptor CCR5, and NK activation receptors (NKp44 and CD16) is represented by colored bars attached to spheres; bar size corresponds to the percentage of cells expressing the receptors among each cell type. Profiling of cytokines for each penile tissue was also performed and is represented by a sprinkled bars colored according to their cognate receptor or functions in red (IL-4, IL-13, and TRAIL), orange (MCP-1), purple (MIP-3α), blue (CCL25), magenta (CCL28), and green (RANTES) (upper-left corner of each scheme region).

Overall, as schematized in Figure 9, we show here that the human penis resembles other mucosal immunological tissues. Concerning T cell subsets, their relative proportions ressemble that of the foreskin (29) and ectocervix (57) but also non-genital tissues (41). B cells relative proportion is also similar to that of other immunologically active mucosa such as the FRT (57), but clearly different from that of the GIT (58) where B cells represent one quarter of the total immune cells. Furthermore, if penile NK cells are phenotypically similar to those found in the lungs, they approximate in proportion to those found in the cervix but are much less abundant than in the endometrium (57). Finally, taken the cytokine/chemokine environment together with chemokine receptors we studied here, CCR10 the best expressed one together with CCR6, CCR3, and CCR9 might participate in the homing of penile immune cells. Differential chemokine receptor expression in each penile compartment suggests that each specific combination of these receptors contributes to specific homing to each penile region. Of note, the frequency of CCR6 and CCR10 expression is always greater than that of the two other receptors, in particular for NK cells.

In conclusion, the human male urethra and the fossa exhibit an immunological composition close to those of other mucosal effector sites, as the foreskin (29) or the FRT (57). Strikingly, the glans shows effector features superior to that of the urethra and the fossa, due to the presence of more activated NK cells and the presence of more terminally differentiate effector CD8^+^ T cells. Thus, one might suggest that the glans is a site more sensitive to an infection such as HIV, whereas the two other more inner penile compartments, namely, fossa and urethra, allow for protection due of their specific immune composition. Nevertheless, urethra, fossa, and glans contain all the components necessary to host an innate immune response with the presence of activated NK cells, as well as an effector adaptive immune response due to the presence of PCs, memory B, and EM T cells. However, our results suggest that the induction of B and T cell responses most likely does not occur in these tissues but rather in draining lymph node, since lymphoid follicles are absent in the penis (6).

Altogether from the present study, the human penis emerges as an efficient mucosal effector site, which contains cell subsets required to induce and generate specific and effective immune responses against mucosal pathogens. This study provides an invaluable source of information on the penile immune system that might contribute to elaborating vaccine strategies against STIs targeting the male genital tract, such as HIV-1.

## Ethics Statement

The study was performed according to local ethical regulations, following approval by the local ethical committee [Comité de Protection des Personnes (CPP) Île-de-France XI, approval no. 11016]. All subjects gave written informed consent in accordance with the Declaration of Helsinki.

## Author Contributions

AS, FR, YG, and MB conceived and designed the experiments; AS, FR, MD, SH, and DD performed the experiments; AS, FR, and MB analyzed data and wrote the article; MR and SC enrolled patients and peformed the surgery.

## Conflict of Interest Statement

The authors declare that the research was conducted in the absence of any commercial or financial relationships that could be construed as a potential conflict of interest.
