# Supplementary material for: The Human Penis Is a Genuine Immunological Effector Site
Source: Front Immunol. 2017 Dec 14;8:1732. doi: 10.3389/fimmu.2017.01732 (PMC5735067; doi:10.3389/fimmu.2017.01732)
Supplement: Supplementary file 1 [file Table_1.PDF]

Target population	Sub-population	Antigen	Clone	Fluorochrome	Reference	Manufacturer	Dilution used
B cells	Identification	CD45	HI30	Alexa Fluor 700	560566	BD	1:50
		CD3	UCHT1	Pacific Blue	558117	BD	1:50
		CD19	HIB19	APC-H7	560727	BD	1:20
	Memory Status	CD21	B-ly4	Pe-Cy7	561374	BD	1:20
		CD27	M-T271	Pe-CF594	562324	BD	1:20
	Plasma cells	CD38	HIT2	PE	555460	BD	1:20
		CD138	MI15	APC	347216	BD	1:20
	Ig receptors	FcRL4	413D12	APC	340206	Biolegend	1:20
		FcRL5	509f6	PE	340304	Biolegend	1:20
T cells	Identification	CD45	HI30	Alexa Fluor 700	560566	BD	1:50
		CD3	UCHT1	Pacific Blue	558117	BD	1:50
		CD4	RPA-T4	APC	555349	BD	1:50
		CD8	SK1	APC	345775	BD	1:50
	Memory Status	CD45RA	HI100	Pe-CF594	562298	BD	1:20
		CD27	M-T271	APC-H7	560223	BD	1:20
		CD62L	LT-TD180	PE	21279624	Immunotools	1:20
		CCR7	3D12	Pe-Cy7	557648	BD	1:20
	Activation	CD38	HIT2	PE	555460	BD	1:20
		CD69	FN50	FITC	555530	BD	1:20
		HLA-DR	G46-6	APC-H7	561358	BD	1:20
	Homing Receptors	CCR3	5^E8	Pe-CF594	562571	BD	1:20
		CCR5	2D7/CCR5	FITC	555992	BD	1:20
		CCR6	11A9	Brillant Violet 605	562724	BD	1:20
		CCR9	112509	Alexa Fluor 488	561608	BD	1:20
		CCR10	1B5	PE	563656	BD	1:20
		CD103	Ber-ACT8	FITC	550259	BD	1:20
		CXCR4	12G5	PE	555974	BD	1:20
	Cytokines	IFNγ	B27	FITC	552887	BD	1:5
		TNF	Mab11	PE	559321	BD	1:5
		IL-2	MQ1-17H12	Pe-Cy7	560707	BD	1:20
		IL-4	MP4-25D2	FITC	554484	BD	1:20
		IL-5	TRFK5	PE	554395	BD	1:20
		IL-17	Ebio64DEC17	FITC	11-7179-42	eBioscience	1:20
		IL-22	22URTI	PercP-eFluor 710	46-7229-41	eBioscience	1:20
NK cells	Identification	CD45	HI30	Alexa Fluor 700	560566	BD	1:50
		CD3	UCHT1	Pacific Blue	558117	BD	1:50
		CD56	B159	Pe-Cy7	557747	BD	1:20
	ADCC	CD16	3G8	APC-H7	560195	BD	1:20
	NK Receptors	NKG2A	Z199	APC	A60797	BC	1:20
		NKG2D	ON72	PE	A08934	BC	1:20
		NKp30	Z25	PE	IM3709	BC	1:20
		NKp44	Z231	PE	IM3710	BC	1:20
		NKp46	BAB281	PE	IM3711	BC	1:20
	Cytokines	IL-17	Ebio64DEC17	FITC	11-7179-42	eBioscience	1:20
		IL-22	22URTI	PercP-eFluor 710	46-7229-41	eBioscience	1:20

Table S1: List of antibodies used for flow cytometry experiments

Fluorochrome	Isotype	Species	Clone	Reference	Manufacturer	Dilution used
FITC	IgG1	Mouse	679.1Mc7	A07795	BD	1:5
	IgG1κ	Mouse	P3.6.2.8.1	11-4714	eBioscience	1:20
	IgG2aκ	Mouse	eBM2a	11-4724	eBioscience	1:20
	IgG1κ	Mouse	MOPC-21	556649	BD	1:5
	IgG1κ	Rat	R3-34	554684	BD	1:5
Alexa Fluor 488	IgG2a	Mouse	G155-178	557703	BD	1:20
PE	IgG1	Mouse	679.1Mc7	A07796	BC	1:5
	IgG1κ	Rat	R3-34	554685	BD	1:5
	IgG2aκ	Mouse	G155-178	554648	BD	1:20
	IgG1κ	Mouse	MOPC-21	556749	BD	1:5
Pe-CF594	IgG1κ	Mouse	X40	562292	BD	1:20
	IgG2bκ	Mouse	27-35	562305	BD	1:20
PerCP-eFluor710	IgG1κ	Mouse	P3.6.2.1	46-4714	eBioscience	1:20
Pe-Cy7	IgG1κ	Mouse	MOPC-21	557872	BD	1:20
	IgG2aκ	Rat	R35-95	552784	BD	1:20
APC	IgG1κ	Mouse	MOPC-21	550854	BD	1:5
APC-H7	IgG2aκ	Mouse	G155-178	560897	BD	1:20
Alexa Fluor 700	IgG1κ	Mouse	MOPC-21	557882	BD	1:20
Pacific Blue	IgG1κ	Mouse	MOPC-21	558120	BD	1:20
Brillant Violet 605	IgG1κ	Mouse	X40	562652	BD	1:20

Table S2: List of isotype controls used for flow cytometry experiments

Cell population	Antigen	Work concentration	Clone	Manufacturer	Reference
Plasma cells	CD138 / Syndecan-1	10 µg/ml	polyclonal	Cymbus	CBL588
T cells	CD3	10 µg/ml	polyclonal	Dako	A0452
	CD8	1.57 µg/ml	C8/144B	Dako	M7103
NK cells	CD56	9.2 mg/ml	123C3	Dako	M7304
Isotypes	Mouse IgG1	1.57 µg/ml<br>9.2 mg/ml	polyclonal	Becton Dickinson	349040
	Rabbit IgG	10 µg/ml	polyclonal	Sigma	12-370

Table S3 : List of antibodies used for immunohistochemistry experiments
